# Evolution towards the elimination of congenital syphilis in Latin America and the Caribbean: a multicountry analysis

**DOI:** 10.26633/RPSP.2019.31

**Published:** 2019-03-15

**Authors:** Mariangela F. Silveira, Rodolfo Gomez Ponce de Leon, Francisco Becerra, Suzanne J. Serruya

**Affiliations:** 1 Women and Reproductive Health Women and Reproductive Health Latin American Center for Perinatology Montevideo Uruguay Latin American Center for Perinatology, Women and Reproductive Health, Montevideo, Uruguay.; 2 Pan American Health Organization Pan American Health Organization Washington, D.C. United States of America Pan American Health Organization, Washington, D.C., United States of America.

**Keywords:** Syphilis, congenital, health promotion, infectious disease transmission, vertical, Latin America, West Indies, Guyana, Sífilis congénita, promoción de la salud, transmisión vertical de enfermedad infecciosa, América Latina, Indias Occidentales, Guyana, Sífilis congênita, promoção da saúde, transmissão vertical de doença infecciosa, América Latina, Índias Ocidentais, Guiana

## Abstract

**Objective.:**

Effective and low-cost interventions for preventing the vertical transmission of syphilis can substantially reduce mortality and morbidity related to maternal and congenital syphilis. This study aims to identify successes and problems in eliminating congenital syphilis in Latin America and the Caribbean (LAC).

**Methods.:**

Conducted in 2015, this multicountry study included qualitative data from focal point staff members of the Pan American Health Organization, as well as country information and answers to semiqualitative questions on the elimination of congenital syphilis. Additional information was obtained from five Caribbean countries and Panama.

**Results.:**

Few of the studied LAC countries use a rapid syphilis test, but most of them do have benzathine penicillin available in primary care facilities. The majority of the countries have national strategies and protocols for eliminating congenital syphilis. There were substantial differences among the national information systems, including with data collection, analysis, and quality control. The major challenges related to eliminating congenital syphilis are the need to improve: prenatal care; test coverage; health worker training about syphilis diagnosis, treatment, and follow-up; and access to institutional deliveries. Other problems include a lack of rapid tests; shortages of benzathine penicillin; and substandard laboratory quality. Poor follow-up of maternal syphilis cases and their sexual contacts was also reported.

**Conclusions.:**

Most of the LAC countries studied have national strategic plans and protocols and have advanced in the elimination of congenital syphilis. These countries must keep improving their capacity to collect high-quality data about coverage and inequities and use this data as a basis for decision-making. To accelerate the elimination of congenital syphilis, the good practices and actions that have been undertaken must be reinforced.

## RESUMEN

**Objetivo.:**

Hay intervenciones eficaces y de bajo costo para prevenir la transmisión maternofetal de la sífilis, que pueden reducir sustancialmente la mortalidad y la morbilidad relacionadas con la sífilis materna y congénita. Este estudio procura identificar los logros y los problemas para eliminar la sífilis congénita en América Latina y el Caribe (ALC).

**Métodos.:**

Este estudio multinacional, realizado en el 2015, incluyó datos cualitativos proporcionados por funcionarios de los centros nacionales de enlace de la Organización Panamericana de la Salud, así como información de los países y las respuestas a preguntas semicualitativas sobre la eliminación de la sífilis congénita. Se obtuvo información adicional de cinco países del Caribe y Panamá.

**Resultados.:**

En pocos de los países de América Latina y el Caribe estudiados se aplica una prueba rápida de sífilis, pero en la mayoría de ellos se cuenta con bencilpenicilina benzatínica en los establecimientos de atención primaria. La mayoría de los países tienen estrategias y protocolos nacionales para eliminar la sífilis congénita. Hubo diferencias significativas entre los sistemas nacionales de información, relacionadas entre otros aspectos con la recopilación, el análisis y el control de calidad de los datos. El principal desafío que conlleva la eliminación de la sífilis congénita es la necesidad de mejorar la atención prenatal, la cobertura de las pruebas, la capacitación del personal de salud en materia de diagnóstico de la sífilis, el tratamiento y el seguimiento, y el acceso a los partos en establecimientos de salud. Otros problemas son la falta de pruebas rápidas, la escasez de bencilpenicilina benzatínica y una deficiente calidad de los laboratorios. También se señaló el seguimiento inadecuado de las pacientes con sífilis materna y sus contactos sexuales.

**Conclusiones.:**

La mayoría de los países de América Latina y el Caribe estudiados tienen planes estratégicos y protocolos nacionales y han avanzado en la eliminación de la sífilis congénita. Estos países deben seguir mejorando su capacidad de recopilar datos de calidad acerca de la cobertura y las inequidades, y usar tales datos como base en la toma de decisiones. Para acelerar la eliminación de la sífilis congénita, deben reforzarse las prácticas y acciones adecuadas que se han emprendido.

## RESUMO

**Objetivo.:**

Intervenções efetivas de baixo custo para prevenção da transmissão vertical de sífilis podem reduzir consideravelmente a mortalidade e a morbidade relacionadas à sífilis materna e congênita. Este estudo visou identificar os bons resultados e os problemas relacionados à eliminação da sífilis congênita na América Latina e no Caribe (ALC).

**Métodos.:**

Estudo plurinacional realizado em 2015 que examinou dados qualitativos obtidos do pessoal dos centros de coordenação da Organização Pan-Americana da Saúde (OPAS), informações fornecidas pelos responsáveis nos países e respostas dadas em um questionário semiqualitativo sobre a eliminação da sífilis congênita. Outros dados foram obtidos de cinco países do Caribe e do Panamá.

**Resultados.:**

Um pequeno número dos países estudados da ALC faz uso do teste rápido para sífilis, mas a maioria dispõe de penicilina benzatina nos serviços de atenção primária. Muitos países têm estratégias e protocolos nacionais para eliminação da sífilis congênita. Verificaram-se diferenças consideráveis entre os sistemas nacionais de informação, inclusive no que se refere à coleta, análise e controle da qualidade dos dados. Os principais desafios relacionados à eliminação da sífilis congênita são a necessidade de melhorar a assistência pré-natal; cobertura do teste; capacitação dos profissionais da saúde no diagnóstico, tratamento e acompanhamento da sífilis e acesso ao parto institucional. Outros problemas são a falta de testes rápidos; desabastecimento dos estoques de penicilina benzatina e qualidade laboratorial abaixo do padrão. Observou-se também o acompanhamento deficiente dos casos de sífilis materna e dos respectivos contatos sexuais.

**Conclusões.:**

A maioria dos países estudados da ALC tem planos e protocolos estratégicos nacionais e fez progresso na eliminação de sífilis congênita. Eles precisam continuar melhorando a capacidade de coleta de dados de alta qualidade relativos à cobertura e iniquidades e usar estes dados para fundamentar a tomada de decisão. Para acelerar a eliminação da sífilis congênita, devem ser reforçadas as boas práticas e as ações realizadas.

The harmful role of maternal syphilis in fetal and newborn health is well known and includes miscarriages, stillbirths, and several types of morbidity that can lead to disability or death. Effective and low-cost interventions for preventing mother-to-child transmission (MTCT) of syphilis, including screening and treatment, can substantially reduce mortality and morbidity related to maternal and congenital syphilis (1).

According to World Health Organization (WHO) global estimates for 2012, Latin America and the Caribbean had the third-highest estimated prevalence of maternal syphilis in the world (0.42%) (2), after the Africa region (1.68%) and the Eastern Mediterranean region (0.57%) (3). For HIV, between 2010 and 2016, Latin America presented a stable trend in new infections among adults, while the Caribbean had a 5% decline. The incidence of HIV among children in Latin America diminished by 60% over that same period (4).

In 2010, the Pan American Health Organization (PAHO) Member States approved the Strategy and Plan of Action for the Elimination of Mother-to-Child Transmission of HIV and Congenital Syphilis, with the goal of eliminating this form of transmission in the Americas by 2015 (5). This initiative for the elimination of mother-to-child transmission (EMTCT) aimed to strengthen surveillance systems for maternal and congenital syphilis; improve use of a PAHO tool called the Perinatal Information System (SIP) in operational research; strengthen the capacity for epidemiological analyses and decision-making; and maximize synergies in actions among the countries of the Americas (5). (Intended to improve the quality of care for mothers and newborns, SIP consists of a group of instruments originally designed for use in obstetrics/gynecology and neonatal services, including the perinatal clinical record, perinatal card, abortion form, partogram, neonatal hospitalization, neonatal nursing, and local data capture and processing programs. Data on each pregnant woman and her child are now collected beginning with the first prenatal visit and are accumulated over successive events until the mother and infant are discharged after delivery. Since data can be used to produce local, regional, and national reports, SIP is a useful instrument for monitoring maternal and neonatal events and evaluating national and regional programs (6)).

In June 2015, WHO announced that Cuba was the first country in the world to have achieved EMTCT of HIV and syphilis (7). This was followed in 2017 by Anguilla, Antigua and Barbuda, Bermuda, the Cayman Islands, Montserrat, and Saint Kitts and Nevis (8).

It is estimated that maternal and congenital syphilis decreased worldwide between 2008 and 2012, suggesting progress towards EMTCT of syphilis. However, untreated maternal syphilis remains a substantial cause of preventable perinatal morbidity and mortality. Among the needed measures to tighten control of mother-to-child transmission (MTCT) of syphilis are: improving access to quality antenatal care, increasing the rate of syphilis testing at the ﬁrst antenatal care visit, ensuring adequate and prompt treatment for women and their partners, and expanding programs of targeted interventions for high-risk groups. Additionally, nationally representative data on core indicators are important for understanding global, regional, and country-level progress in eliminating this important public health problem (3).

The 2015 PAHO update on EMTCT of HIV and syphilis in the Americas reported that, while the percentage of pregnant women with HIV who receive antiretrovirals increased, the percentage of pregnant women with syphilis who received treatment remained stable, with an average of 85% in 2014. The report states that progress towards EMTCT has proceeded more rapidly for HIV than for syphilis. The PAHO report also mentioned the existence of gaps in data quality, mainly in the detection and treatment of maternal syphilis and the diagnosis and reporting of congenital syphilis (9).

This study aims to identify successes and problems in eliminating congenital syphilis in the countries of Latin America and the Caribbean (LAC).

## MATERIALS AND METHODS

This report is based on a multinational study that includes qualitative data provided by PAHO EMTCT focal point staff members from LAC countries in 2015, as well as on additional information on five Caribbean countries and Panama. The study includes information on: 1) existence of a national strategic plan for EMTCT of HIV and congenital syphilis; 2) maternal and child health and sexual and reproductive health programs; 3) updated guidelines and protocols regarding EMTCT; and 4) status of national health information systems that allow analysis of progress towards EMTCT of congenital syphilis, SIP information coverage on a national level, and the availability of a rapid syphilis test and benzathine penicillin in primary care.

The study included semiqualitative questions evaluating the following changes in the preceding three years: 1) changes in information systems that allow EMTCT indicators to be examined more quickly and accurately; 2) changes that support EMTCT of congenital syphilis; 3) major remaining difficulties that prevent EMTCT of HIV and congenital syphilis; 4) actions and current developments in each country toward EMTCT of HIV and congenital syphilis; and 5) information gaps, including with lost or absent data and with data on such items as ethnicity, education, parity, access to quality prenatal care, and planned pregnancy.

For the study, additional information was obtained on five Caribbean countries (Dominica, Grenada, Saint Lucia, Saint Vincent and the Grenadines, and the Turks and Caicos Islands) from two sources: 1) the 2016 EMTCT cluster report for the Eastern Caribbean Countries (ECC) and 2) the 2016 cluster report on the United Kingdom Overseas Territories. For Panama, information was extracted from the country’s 2016 EMTCT report.

Since the study utilized only secondary and published data, a submission to an ethics committee was not required.

## RESULTS

This report presents results from 14 countries that participated in the study: Argentina, the British Virgin Islands, Chile, the Dominican Republic, El Salvador, Guatemala, Guyana, Haiti, Honduras, Paraguay, Peru, Trinidad and Tobago, Uruguay, and Venezuela. In addition, information on the four ECC countries (Dominica, Grenada, Saint Lucia, and Saint Vincent and the Grenadines), the Turks and Caicos Islands, and Panama was obtained from their 2016 cluster reports. Nine countries did not answer the request for information, despite several contacts from the researchers.

Seven countries (Guatemala, Guyana, Haiti, Peru, Trinidad and Tobago, the Turks and Caicos Islands, and Venezuela) do not use the SIP tool. SIP coverage in Argentina was 72.2% of births; in Chile it was 80% in the public and private health systems, and an electronic version was also being implemented. Although SIP is not implemented on a national level in the Dominican Republic, it is being used in five main maternity hospitals. El Salvador has implemented SIP in 28 public maternity hospitals, covering 85% of the population. In Honduras, the SIP coverage is 45%, and with problems in updating the information. ECC countries utilize SIP in all maternal and child health care clinics. Panama also uses SIP, although with incomplete data, and Paraguay’s national SIP coverage was 13.6%. Uruguay utilizes SIP in all health facilities, with 96.6% coverage of the births in 2015.

Rapid syphilis testing is available in primary care facilities in the British Virgin Islands, Guatemala, Haiti, Honduras, Paraguay, Peru, and Uruguay, but not in Chile, the Dominican Republic, El Salvador, Panama, Trinidad and Tobago, the Turks and Caicos Islands, and Venezuela. No information was available for Argentina, Guyana, or the four ECC countries.

Benzathine penicillin is available in primary care in the British Virgin Islands, Chile, the Dominican Republic, El Salvador, Guatemala, Haiti, Panama, Paraguay, Peru, and Uruguay. No information was available for Argentina, the ECC countries, Guyana, Honduras, Trinidad and Tobago, the Turks and Caicos Islands, and Venezuela.

Table 1 summarizes the present situation regarding the EMTCT of HIV and syphilis in 20 LAC countries, with this and several following paragraphs providing additional details on conditions in each nation. The Dominican Republic reported developing a new national elimination plan for 2017-2021. Under its 2017-2022 National Strategic Plan for Sexual and Reproductive Health, Haiti will integrate the MTCT of HIV and syphilis. El Salvador and Guatemala are in the process of updating their guidelines and protocols with respect to the EMTCT of HIV and syphilis.

Regarding the availability of data for tracking the elimination of congenital syphilis, although the Dominican Republic collects data on the necessary variables, there are problems with data collection and flow. In addition, El Salvador needs to enforce full completion of perinatal forms and case closures in its surveillance system.

There is a lack of uniformity among the national information systems in the LAC countries studied. Compulsory notification of events in Argentina’s National Health Surveillance System includes maternal and congenital syphilis cases, and SIP is used for test coverage. In the British Virgin Islands, private care doctors and services must report on communicable diseases to the Ministry of Health. Chile’s sources of information are based on universal case surveillance and periodic progress reports by care centers. In the Dominican Republic, several systems collect information related to EMTCT, but there is not a unified information system, leading to discontinuities and difficulties in accessing EMTCT indicators.

El Salvador has a central information system that includes an epidemiological surveillance system, morbidity and mortality information, and information from laboratories, hospitals, and outpatient clinics. In Guyana, data collected on HIV and syphilis is entered into the antenatal care/EMTCT register at health centers, with a monthly summary produced in the Department of Statistics.

In Haiti, individual information is collected through electronic medical records systems. At clinics specialized in preventing MTCT of HIV and syphilis, nurses are trained to ensure completion of case reports. Honduras’ information system is not working properly due to information flow issues, analysis problems, and the nonutilization of available information.

Dominica, Saint Lucia, and Saint Vincent and the Grenadines have MTCT prevention coordinators who support all HIV-positive pregnant women and collect data on pregnant women from primary care clinics. In Grenada, public health nurses take care of HIV-positive pregnant women and follow up with exposed infants after birth. Efforts have been made in the Turks and Caicos Islands to integrate HIV services into primary health care and sexual and reproductive health care. Although Panama has a National Information System (SIS), a National Surveillance System, a Laboratory Information System (SILAB), and SIP, there are problems integrating the systems and maintaining data quality. Paraguay is expanding its Experto health information system and is planning to improve SIP quality. Peru collects information through several systems. Uruguay uses data from SIP, live birth certificates, the DEVISA surveillance system, and MTCT case studies. In Trinidad and Tobago, key people responsible for syphilis data in the country use paper-based data collection methods.

**TABLE 1:** Situation with the elimination of mother-to-child transmission (EMTCT) of HIV and congenital syphilis in countries of Latin America and the Caribbean, as of 2014–2016

Country	National strategic plan for the elimination of mother-to-child transmission of HIV and congenital syphilis (CS)	Linkage to MCH^a^ and SRH^b^ programs	Guidelines and protocols on PMTCT^c^	Collection of data sufficient to analyze CS elimination	National information system
Argentina	Yes	Yes	Yes	Yes	Yes
British Virgin Islands	Yes	NR^d^	NR	NR	Yes
Chile	Yes	Yes	Yes	Yes	Yes
Dominica, Grenada, St. Lucia, and St. Vincent and the Grenadines	Yes	Yes	Yes	Yes	Yes
Dominican Republic	Yes, 2011–2015	NR	Yes, 2014	Yes	Yes
El Salvador	Yes, 2015	Yes	Yes, 2015/2016	Yes	Yes
Guatemala	Yes, 2012–2016	Yes	Yes, 2013	NR	NR
Guyana	Yes	NR	Yes	Yes	Yes
Haiti	Yes, 2010–2015	No	Yes	No	No
Honduras	Yes	No	NR	No	No
Panama	Yes, 2014	Yes	Yes	Yes	Yes
Paraguay	Yes, 2014	Yes	Yes	Yes	Yes
Peru	Yes, 2017–2021	Yes	Yes	Yes	Yes
Trinidad and Tobago	No	NR	Yes, 2014 (in draft and U.S. Centers for Disease Control and Prevention guidelines)	Yes	No
Turks and Caicos Islands	Yes	Yes	Yes	Yes	Yes
Uruguay	Yes, 2015	Yes	Yes	Yes	Yes
Venezuela	No	No	Yes	No	Yes

***Source:*** Prepared by the authors, based on the study results.

a MCH = maternal and child health.

b SRH = sexual and reproductive health.

c PMTCT = prevention of mother-to-child transmission.

d NR = not reported.

Table 2 and the remaining paragraphs of the Results section aim to reflect changes in national information systems to obtain MTCT indicators quickly and accurately, changes towards the EMTCT of congenital syphilis (CS) and HIV, and difficulties in and actions to achieve EMTCT of HIV and CS. For example, Paraguay has made changes to more quickly obtain better information, focusing on mandatory notification, health worker training, and expanding the Experto system. To obtain data, Trinidad and Tobago started to use variables from its antenatal care program, such as on syphilis diagnosis, treatment, and care. Uruguay has instituted congenital syphilis case audits, allowing data to be compared through surveillance programs to present more reliable figures.

**TABLE 2:** Changes and challenges for the elimination of mother-to-child transmission (EMTCT) of HIV and congenital syphilis (CS) in countries of Latin America and the Caribbean, 2014-2016

Country	Changes in information systems, to obtain MTCT indicators quickly and accurately	Significant changes towards the EMTCT of CS and HIV	Continuing difficulties in achieving EMTCT of HIV and CS	Actions and national developments to reach EMTCT of HIV and CS
Argentina	Improved surveillance increasing reports of MS^a^ and CS cases	NR^b^	Lack of personnel; unclear definitions of responsibilities for MS case active follow-up; need to scale up access to timely diagnosis and treatment; lack of CS case audits	Evaluating the possibility of monitoring jurisdictions’ compliance with the targets; reinforcing guideline use and staff training
British Virgin Islands	Amended registers to capture testing at the ANC^c^ and delivery sites	EMTCT validation exercise in 2016	Need to upgrade laboratory quality	Efforts under way to achieve accreditation and implement quality assurance program for the national laboratory
Chile	Implemented a monitoring system of EMTCT process indicators	Prioritized EMTCT at all levels of health care	Need to implement a second HIV test in pregnancy and sexual partner testing; need to improve primary prevention strategies	National strategy for EMTCT, including case studies; improvement plan for critical nodes, surveillance system, and registries
Dominica, Grenada, St. Lucia, and St. Vincent and the Grenadines	NA^d^	NA	NA	NA
Dominican Republic	Integrated syphilis-related variables within HIV database	Reexamining surveillance and control of MS	The process of reform in the health system is a temporary obstacle to EMTCT; need for training on syphilis diagnosis and treatment	Developing a national strategy on EMTCT that prioritizes challenges
El Salvador	Resumed SIP as a tool for provision of ANC and delivery in the public health system; established platforms for real-time monitoring of HIV-positive pregnant women and exposed children	Established a monitoring system to close cases of HIV-exposed children	Need to implement case audits and SIP Web; need to improve test coverage and data collection and availability	Developing MS and CS care cascades; joint work among health programs; an intersectoral panel for EMTCT
Guatemala	No changes	NR	Difficulties in improving ANC and testing coverage; underreporting of cases	RT^e^ for syphilis and HIV in primary care
Guyana	Reviewed EMTCT protocols and mechanisms for data processing, analysis, and dissemination	NR	Need to enforce use of unique identifiers; need to report syphilis treatment; need to improve data on live births and ANC coverage	NR
Haiti	Increased health facilities’ reporting on EMTCT	Evaluated progress on EMTCT of HIV and CS; implemented a road map towards elimination in 2020	Low access to institutional deliveries; confirmation tests not available; problems in screening and management of pediatric cases; lack of follow-up with MS cases and sexual contacts; weak management of laboratory data; shortages of benzathine penicillin	Implemented a road map on EMTCT
Honduras	No changes	A new organization model, with decentralization of health services, had a negative impact on the information system	No information and monitoring on the EMTCT indicators; suboptimal coverage of screening in pregnancy and newborns	Updating and extending SIP with a national platform
Panama	NR	NR	Need to extend and qualify ANC; need to increase RT syphilis use in primary care; need to improve SIP data quality; need to avoid supply shortages	Intensifying efforts on EMTCT; implementation of SIP Web; training health workers; reinforcing MS and CS surveillance
Paraguay	Mandatory notification of MTCT cases; health worker training; Experto system expansion	Prioritized EMTCT of HIV and CS, through broader use of RTs, availability of benzathine penicillin, and health worker training; updated national guidelines	Low coverage of ANC and syphilis testing in pregnancy; late start of ANC; lost opportunities to access and test pregnant women at first visit; low adherence to syphilis treatment guidelines	Strategic Plan for HIV/STIs 2014-2018; Reproductive and Sexual Health Plan 2014-2018 integrates EMTCT; Adolescent Health Plan; new guidelines for EMTCT
Peru	Implemented new policy for epidemiological surveillance of HIV and STIs	Included notification of MS and MTCT of HIV and CS cases; improved nominal identification of HIV and MS cases and exposed children; individual monitoring at regional and local level, especially in marginal and rural areas; updated diagnostic algorithms for HIV and syphilis	Major problems in poor and rural areas	NR
Trinidad and Tobago	Started to use variables included in its ANC program (syphilis diagnosis, treatment, and care)	Enhanced surveillance activities, including with contact tracers to find cases	Logistics issues; late referral of MS cases for treatment; staffing issues; need to implement SIP at all levels of the program	Positive changes in political will; commitment by health care providers; community awareness; increasing coverage of HIV and syphilis testing
Turks and Caicos Islands	NA	NA	NA	NA
Uruguay	Instituted CS case audits	NR	NR	NR
Venezuela	No changes	NR	Lack of RT; suboptimal ANC coverage; low engagement of primary care team with EMTCT	Incorporation of RT for syphilis and HIV; national plan for EMTCT

***Source:*** Prepared by the authors, based on the study results.

a MS = maternal syphilis.

b NR = not reported.

c ANC = antenatal care.

d NA = not available.

e RT = rapid test.

Significant changes towards the EMTCT of congenital syphilis and HIV are being implemented in Latin America and the Caribbean. For instance, Chile has made this issue a priority at all levels of health care. The British Virgin Islands performed a validation exercise in 2016. The Dominican Republic is reexamining surveillance and control of maternal syphilis. El Salvador has established a monitoring system to close cases of HIV-exposed children. Haiti evaluated its progress toward EMTCT of HIV and congenital syphilis with a road map to achieve that by 2020. Paraguay has prioritized EMTCT of HIV and congenital syphilis in its public health policies through more extensive use of rapid tests (RTs), increased availability of benzathine penicillin and health worker training, and updated national guidelines. Peru has implemented a new epidemiological surveillance policy for HIV and sexually transmitted infections, including notification of maternal syphilis and the MTCT of HIV and syphilis cases. Trinidad and Tobago has enhanced its surveillance activities, including employment of contact tracers to find cases and bring them for treatment.

There are many major perceived difficulties in achieving EMTCT of HIV and congenital syphilis in Latin America and the Caribbean. For example, in Argentina, these challenges include a lack of personnel, unclear definitions of responsibilities in the active follow-up of maternal syphilis cases, a need to increase access to timely diagnosis and treatment, and a lack of congenital syphilis case audits. In Guatemala, there are difficulties with improving antenatal care and test coverage, as well as substantial underreporting of cases. Guyana needs to enforce the use of unique identifiers to provide better information on testing, include syphilis treatment in the surveillance report, and improve data on live births and antenatal care coverage. In Panama, there is a need to extend and qualify antenatal care (especially among vulnerable populations), to increase use of rapid syphilis testing in primary care, and to collect quality SIP data for EMTCT indicators, thus allowing timely decision-making and data triangulation and avoiding supply shortages.

There are a number of current actions and national developments toward EMTCT of HIV and congenital syphilis in Latin America and the Caribbean. Argentina is evaluating the possibility of monitoring jurisdictional compliance with elimination goals. In the British Virgin Islands, efforts are under way to achieve accreditation and implement a quality assurance program for the national laboratory. Chile has a national EMTCT strategy that includes condom use promotion, case studies, and a plan to improve surveillance systems and registries. The Dominican Republic is developing a national EMTCT strategy that prioritizes the challenges mentioned in its midterm evaluation and recent studies. El Salvador is developing maternal and congenital syphilis care cascades, cooperation between health programs, and an interdisciplinary panel on EMTCT. Guatemala started using rapid syphilis and HIV tests in primary care. Honduras is updating and extending SIP to all hospitals for a national platform with real-time information systems that will contribute to decision-making at different levels. Panama is intensifying efforts towards EMTCT, including implementation of a SIP network, health personnel training, and reinforcement of maternal and congenital syphilis surveillance. Paraguay has its 2014-2018 Strategic Plan for HIV/Sexually Transmitted Infections, its 2014-2018 Reproductive and Sexual Health Plan (which integrates EMTCT), and its Adolescent Health Plan (which guarantees individual access to health services). Trinidad and Tobago reported positive changes in political will and the commitment of health care providers, awareness among citizens about EMTCT, and increasing coverage of HIV and syphilis testing. Venezuela mentioned implementing rapid syphilis and HIV tests and a national EMTCT plan.

In terms of missing SIP data, El Salvador’s average was 3%, with great unevenness among the variables; for Honduras the average was 8%; and in Uruguay at least one of the necessary variables was missing for 11.5% of cases. With respect to missing congenital syphilis indicators data, Haiti and Honduras do not control for it, while in El Salvador the missing data ranges from 0.06% to 66.5%. Stratified analyses of possible inequality indicators such as ethnicity, education, and parity do not occur in Guyana, Haiti, and Honduras, while El Salvador and Uruguay collect information on these variables and can perform equity analyses.

## DISCUSSION

The difficulty in the data collection process differed among the countries we studied. Another study, by Serruya et al. (10), used data from select LAC nations and also found that although most of the countries had a national plan to eliminate congenital syphilis, as well as updated protocols and guidelines for managing and treating maternal and congenital syphilis, the evaluation of the countries’ progress was limited by large amounts of missing data.

LAC countries must continue to expand their capacity to collect high-quality data on intervention coverage and inequities, using it as a basis for decisions on how best to reach women and children (10). WHO guidelines on ethical issues in public health surveillance state that countries have an obligation to develop appropriate, feasible, sustainable public health surveillance systems with a clear purpose and a plan for data collection, analysis, use, and dissemination (11). WHO also emphasizes that strong national systems will form the basis for an effective regional and global network for the surveillance and control of communicable diseases, and that the development and strengthening of national surveillance requires a substantial long-term commitment of human and material resources (12).

Nine countries did not answer our request for information. However, given their distribution in the LAC region and their EMTCT rates, it is unlikely that their results would have been different from the ones included in this study.

Countries in which SIP has been more consistently implemented, such as El Salvador, were able to rapidly produce more complete information for our survey. SIP has been used successfully to monitor maternal and child health in various LAC nations (13, 14).

Control of missing data and control of data related to EMTCT indicators occurs in both El Salvador and Uruguay. Guyana mentioned the need for a unique identifier number to improve information quality. Since El Salvador and Uruguay also have information on inequality indicators, they can thus perform stratified analyses to identify key populations that should be reached in order to hasten EMTCT of HIV and syphilis.

One positive finding is that most of the countries have a national plan for EMTCT of HIV and congenital syphilis, as well as guidelines and protocols in place regarding EMTCT. Moreover, although the availability of rapid syphilis testing in primary care is not widespread, benzathine penicillin is normally available in every country that provided information for our study. This is important, given that the risk of treating pregnant women with benzathine penicillin to prevent congenital syphilis is very low and does not outweigh its benefits (15).

The reported changes in the information systems in the British Virgin Islands, the Dominican Republic, El Salvador, Guyana, Haiti, and Panama should lead to better information on health indicators. This is especially true with respect to EMTCT, as demonstrated by Cuba and its being the first country in the world to achieve EMTCT of HIV and syphilis. Most of the countries in our study reported that their information systems still pose a challenge to achieving EMTCT.

Other countries in our study reported the need for improvements in other areas, including antenatal care and syphilis test coverage, health worker training in syphilis diagnosis, treatment and follow-up (including of newborns and sexual contacts of infected mothers), access to institutional deliveries, availability of rapid syphilis testing and of benzathine penicillin, and laboratory quality. A 2016 survey led by WHO found that five countries in the Americas (Brazil, Jamaica, Panama, Suriname, and Trinidad and Tobago) experienced shortages of benzathine penicillin, and three other nations (Chile, Costa Rica, and Nicaragua) reported problems in purchasing benzathine penicillin (16).

Low engagement of primary care teams with the subject of HIV and syphilis was also reported by some countries. On the other hand, actions and good practices have been undertaken to accelerate EMTCT in some nations. El Salvador mentioned the development of maternal and congenital syphilis care cascades and the linkage between health programs. Honduras is in the process of updating and extending SIP, as well as implementing a real-time information system. The Dominican Republic is developing a national strategy for EMTCT that prioritizes challenges pointed out in recent evaluations. Haiti implemented a road map towards EMTCT. The British Virgin Islands is making efforts to achieve accreditation, especially for their laboratories.

The 2015 PAHO update on the EMTCT of HIV and syphilis (9) found that antenatal care access in Latin America and the Caribbean has improved, but 1.4 million women attended fewer than four antenatal care visits, of which half a million received no antenatal care at all. There are also problems in increasing syphilis testing and treatment, gaps in data collection, and shortfalls in data quality, especially for syphilis (9).

One very positive finding was that there had been a reduction in congenital syphilis cases in all six LAC countries that participated in an evaluation for the 2010–2012 period (10). There has also been a decrease in the proportion of stillbirths attributed to maternal syphilis for most countries with available information. This is in line with the 2015 PAHO update, which lists 11 countries that may have already eliminated the MTCT of syphilis, in addition to 7 nations in which this has already been confirmed, as well as several countries that are currently making progress toward this goal (9).

We can conclude that, in general, EMTCT indicators have improved in LAC countries. It is important to point out that most of the surveyed nations have made an effort to achieve better quality, not only in health services but also in information systems. However, despite a great amount of work from health managers to access the information necessary to demonstrate the extent of progress toward EMTCT, greater efforts are still required.

There is room to improve every health care process in Latin America and the Caribbean aiming to help eliminate congenital syphilis, such as with better access to adequate antenatal care and with health worker training, especially regarding adherence to diagnostic and treatment guidelines. In addition, since information quality plays a substantial role in the validation process, systems such as SIP Web (which is the SIP version available on the Internet) (17) and the Latin American Center for Perinatology, Women and Reproductive Health (CLAP) network (18) (developed by sentinel centers in LAC for surveillance and research on women and maternal and neonatal health) should be supported in their efforts to improve the quality of health information and to allow easier access to data related to EMTCT indicators. Each country must define its own priorities and how best to achieve them in order to accelerate the elimination of congenital syphilis.

## Author contributions.

RGPL and MFS conceived the original idea and collected and analyzed the data. All the authors helped write and review the paper, as well as approved the final version.
